# Innovations and Advances in Schistosome Stem Cell Research

**DOI:** 10.3389/fimmu.2021.599014

**Published:** 2021-03-05

**Authors:** Hong You, Malcolm K. Jones, Deanne J. Whitworth, Donald P. McManus

**Affiliations:** ^1^Department of Immunology, QIMR Berghofer Medical Research Institute, Brisbane, QLD, Australia; ^2^School of Veterinary Science, The University of Queensland, Gatton, QLD, Australia

**Keywords:** schistosomes, helminths, stem cells, neoblasts, germinal cells

## Abstract

Schistosomes infect about 250 million people globally causing the devastating and persistent disease of schistosomiasis. These blood flukes have a complicated life cycle involving alternating infection of freshwater snail intermediate and definitive mammalian hosts. To survive and flourish in these diverse environments, schistosomes transition through a number of distinct life-cycle stages as a result of which they change their body plan in order to quickly adapt to each new environment. Current research suggests that stem cells, present in adults and larvae, are key in aiding schistosomes to facilitate these changes. Given the recent advances in our understanding of schistosome stem cell biology, we review the key roles that two major classes of cells play in the different life cycle stages during intramolluscan and intramammalian development; these include the germinal cells of sporocysts involved in asexual reproduction in molluscan hosts and the neoblasts of adult worms involved in sexual reproduction in human and other mammalian hosts. These studies shed considerable new light in revealing the stem cell heterogeneity driving the propagation of the schistosome life cycle. We also consider the possibility and value of establishing stem cell lines in schistosomes to advance schistosomiasis research. The availability of such self-renewable resources will provide new platforms to study stem cell behavior and regulation, and to address fundamental aspects of schistosome biology, reproductive development and survival. In turn, such studies will create new avenues to unravel individual gene function and to optimize genome-editing processes in blood flukes, which may lead to the design of novel intervention strategies for schistosomiasis.

## Introduction

Schistosomiasis is a devastating disease and a serious public health problem in the developing world, with an estimated 250 million people infected in 78 countries (1, 2). No human vaccine is available (3) and treatment is entirely dependent on a single drug (praziquantel) that is widely used, raising concerns that the development of drug resistance may pose a major hindrance to achieving global control of schistosomiasis in the near future. Furthermore, the COVID-19 pandemic will likely severely impact schistosomiasis and other neglected tropical diseases control programs over the coming years (4). The schistosome lifecycle is complex, consisting of a number of disparate developmental stages involving free-living and parasitic forms in tandem with alternating phases of asexual reproduction in molluscan intermediate hosts and sexual reproduction in human and other definitive mammalian hosts. Unraveling how these blood flukes transition and adapt functionally and reproductively to these different environments will be critical in the identification of new drug and vaccine targets that can provide effective intervention tools to interfere with the transmission of schistosomiasis and to prevent infection and disease.

**Figure 1:**
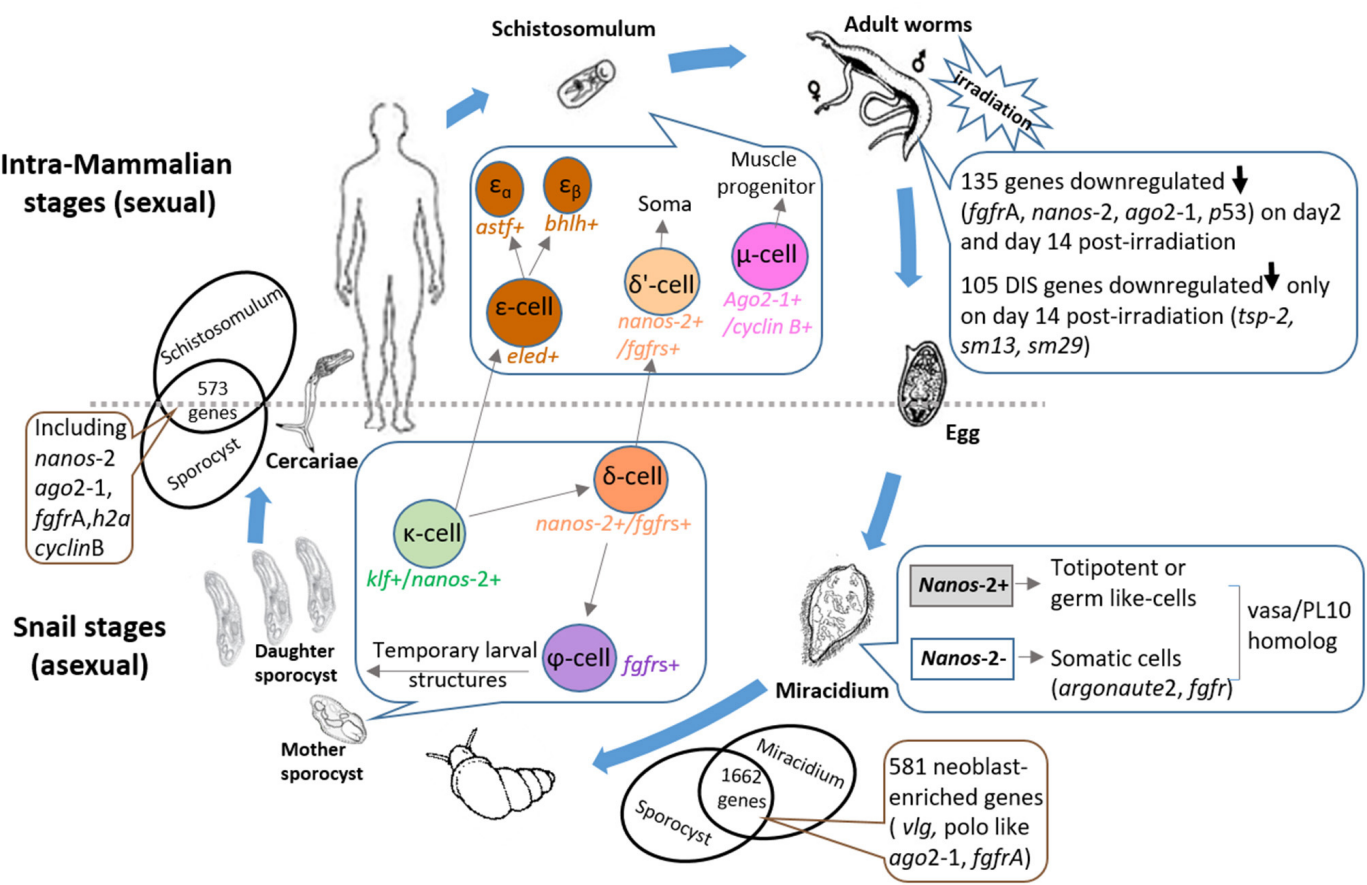
Diagram illustrating the key roles that stem cells play throughout the schistosome life cycle. Snail stages: Eggs released in the excrements of mammalian hosts pass into the external environment and hatch into free-swimming ciliated miracidia in fresh water. Two distinct germinal cell lineages (*nanos*-*2*^+^ and *nanos*-*2*^−^ cells) have been identified in miracidia (5). The miracidium proceeds to infect a suitable snail host leading to a dramatic process of development in which the larva transforms into a mother sporocyst. Of 1662 conserved genes expressed in miracidia and sporocysts 48 hr post-transformation, 581 were shown to share similarity with planarian neoblast-enriched transcripts (5). Three sporocyst stem cell classes were designated: κ-cells (*klf*^+^, *nanos-2*^+^); φ-cells (*fgfrA, B*^+^); and δ-cells (*nanos*-*2*^+^ and *fgfrA, B*^+^) (6). In *in vitro*-transformed mother sporocysts, key genes upregulated in κ-cells include *fgfrA/B, p53* and *zfp-1*, leading to the formation of δ-cells. Downregulation of *nanos-2* and activation of *hesl-2* in δ-cells causes the specification of φ-cells. The germinal cells in the mother sporocyst are able to undergo asexual clonal expansion to release hundreds of daughter sporocysts; these produce more daughters and infective cercariae which escape from the snail intermediate host. Intra-mammalian stages: The free-swimming cercariae penetrate the skin of the mammalian host when they contact water and these larvae transform into schistosomula which enter the host circulation. Mother sporocysts and schistosomula share 573 enriched genes including stem cell markers (e.g., *nanos-2, ago2-1*, and *fgfrA*) (6). Schistosomula stem cells are predicted to be further separated into three main populations: δ'-cells (*nanos*-*2*^+^ and *fgfr*s^+^), ε-cells (*eled*^+^) and μ-cells (*ago2-1*^+^ and *cyclin B*^+^) (7). Activation of *hesl* in δ-cells of the mother sporocyst may lead to δ-cells in the schistosomulum. Downregulation of *nano*s-*2* and activation of *eled* in the κ-cells of mother sporocysts generates ε-cells, which subsequently develop into two subpopulations (6) (ε_α_- and ε_β_-cells). Supported by the differentiation of stem cells, schistosomula grow to adult worms in the definitive host and build up an extensive arrangement of sexual reproductive organs *de novo*. When adult *S. mansoni* were exposed to irradiation, 105 down-regulated genes on day 14 post-irradiation (8) were identified as delayed irradiation-sensitivity (DIS) genes that may encode potential drug/vaccine targets. Paired female worms produce and lay fertilized eggs, many of which become entrapped in tissues evoking inflammatory responses, leading to granuloma formation and hepatic fibrosis. The remainder travel to the intestine or bladder and are released from the host to ensure continuation of the life cycle. *ago*, argonaute; *h2a*, histone 2a; *fgfr*, fibroblast growth factor receptor; *vlg*, vasa-like; *klf*, krüppel-like factor; *zfp, zinc finger protein*; *hesl, hes* family transcription factor; astf, aschaete-scute transcription factor; bhlh, basic helix-loop-helix protein; *tsp*-2, *tetraspanin-2*.

In the schistosome lifecycle, eggs laid by mature female schistosomes are released in excrement (urine, feces) from the definitive host into the external environment and these hatch in fresh water into free-swimming miracidia. These ciliated larvae infect a snail intermediate host and develop into mother sporocysts (Figure 1). Within the sporocysts is a population of totipotent stem cells, called germinal cells, that are thought to drive the asexual clonal expansion of mother sporocysts to give rise to hundreds of daughter sporocysts (5, 9), due to the division of these germinal cells in mother sporocysts producing progeny that have the capacity to independently initiate the embryogenesis of daughter sporocysts. This stage follows a similar pattern of reproduction to the mother sporocyst, generating either new sporocysts, or generation of the next phase involving thousands of infective cercariae. This clonal reproduction process is repeated during the life of an infected snail, producing massive numbers of cercariae that are released into water. Hence, the stem cell-driven processes of proliferation and embryogenesis are of key importance in the propagation of schistosomes. Humans and other mammals are infected via contact with fresh water where contaminating cercariae penetrate the skin. In the skin the parasites transform into schistosomula, which then enter the host's circulation facilitating their migration to the liver where they will mature into adult worms. The juvenile worms build a functional digestive system and develop sexual reproductive organs through *de novo* processes that commence by differentiation of pluripotent stem cells, called neoblasts, early on in schistosomula differentiation (6). The adult parasites can survive long-term for decades (10) in the harsh microenvironment of the blood system of the mammalian host. During this process, neoblasts play necessary roles in somatic tissue renewal, especially the renewal or repair of the tegumental host-parasite interface damaged due to aging or by host immune mechanisms (8); these cells are also involved in the genesis of reproductive tissue (6) in pairing-induced processes (which occurs when the female worm pairs with a male parasite). Female worms after pairing with males produce fertilized eggs, some of which pass to the intestine (*Schistosoma mansoni* and *S. japonicum*) or bladder (*S. haematobium*) and are released into the exterior to complete the life cycle. Many eggs, however, are entrapped in tissues such as the liver, evoking inflammatory responses leading to granuloma formation, hepatic fibrosis, and chronic disease (11).

Successful completion of the schistosome lifecycle relies on the critical roles of two cell populations:

- Germinal cells: defined as “totipotent stem cells” (5, 6, 10), these are able to give rise to embryos; historically called “germinative cells,” they are involved in the clonal expansion of the asexual sporocyst stages in molluscan hosts and play important roles in the transformation and replication process. It has been demonstrated that this proliferative larval cell population of germinal cells (5) shares conserved features of distinct stem cell morphology with stem cells of planarians (close relatives of schistosomes) (12, 13).
- Neoblasts: defined as “adult pluripotent stem cells” (13), these have the ability to develop into specific types of differentiated cells, and they have been identified in adult schistosomes. Two distinct types of stem cell populations (somatic stem cells and germinal cells) have been recently identified in schistosomula (6)—the early intra-mammalian developmental stage; these two cell types are responsible for somatic tissue differentiation and the production of germ cells (sperm and oocytes), respectively, in adult schistosomes in the definitive host. Furthermore, neoblasts promote parasite longevity in the mammalian host (10), playing key roles in development and, ultimately, pathogenesis (14). They maintain the ability of schistosomes to thrive in definitive hosts resulting in chronic inflammation and host immune modulation by affecting inter- and intracellular communication (15).

Improved understanding of the stem cell biology of schistosomes has been aided by studies on the free-living planarian relatives of schistosomes, such as *Schmidtea mediterranea*, which has been used as a valuable model organism (16). In planarians, a population of pluripotent stem cells (neoblasts) are responsible not only for regeneration but are involved also in worm growth and tissue homeostasis (17). The similarity in function and morphology (round-to-ovoid mesenchymal cells with a high nuclear to cytoplasmic ratio (12) of neoblasts in both planarians and schistosomes, and the germinal cells in schistosome sporocysts, implies that these three cell types have likely been highly involved in the evolution of parasitic and free-living flatworms (18).

Nevertheless, many intriguing questions remain unanswered in regards to schistosome stem cells. For instance, how do blood fluke stem cells drive the development and differentiation of each life cycle stage that result in these parasites thriving in diverse and challenging environments? When do neoblasts form during schistosomal embryogenesis? Is the self-renewal ability of these cells exhaustible under host immune or metabolic pressure (19)? Answers to these questions will provide important clues in our understanding the fundamental biological processes governing the propagation and long-term survival of schistosomes in their human hosts (6). To this end we reviewed very recent publications focusing on the important roles played by stem cell populations in critical phases of schistosome development across the multiple alternating generations of the life cycle. The insights gained from studies of these cells may provide key insight into survival and reproduction of schistosomes and other neodermatan parasites, while also opening new pathways of single cell culture and manipulation to screen functions of many uncharacterised schistosome molecules.

## The Key Roles Played by Stem Cells Throughout the Schistosome Life Cycle

### Larval Cell Population (Germinal Cells)

In 1954, Cort et al. (20) hypothesized on the important roles that totipotent germinal cells play throughout the schistosome life cycle and in driving reproduction. Subsequently, Pan (21) showed that these special cells had a stem cell-like morphology and rapidly proliferated in miracidia and sporocysts. However, a major question is how the limited number of germinal cells in each miracidium (5) are able to give rise to such large numbers of daughter sporocysts during the developmental process.

Recently, a range of different methods have been developed that allow study of the schistosome germline including the use of thymidine analog 5-ethynyl-2′-deoxyuridine (EdU) labeling, whole-mount *in situ* hybridization, and RNA interference (22). Using RNA sequencing (RNAseq), Wang et al. (5) compared transcripts enriched in planarian neoblasts with the gene expression profiles of miracidia and mother sporocysts at 48 h post-transformation of miracidia *in vitro*. They demonstrated that there are two distinct germinal cell lineages represented in mother sporocysts at 48 h post-transformation: cells that express the transcription factor *nanos-2* (*nanos-2*^+^) and those that do not (*nanos-2*^−^). *Nanos-2*^+^ germinal cells appeared to be more totipotent or germ cell-like, whereas *nanos-2*^−^ cells may be more primed toward somatic fates. *Nanos-2* appears to play an important role in germline development in nematodes, insects, and vertebrates (23), and has been shown to function in schistosomes as a conserved regulator of germ cells (24) and adult stem cells (13).

Wang et al. (5) also used RNAseq to compare the gene expression profiles of miracidia and sporocysts in an attempt to identify transcripts specific to the germinal cells; 1,662 genes were upregulated in sporocysts compared with miracidia and, of these, 581 genes shared similarity with planarian neoblast-enriched transcripts. Furthermore, these authors used reciprocal BLAST comparisons to examine the expression of orthologous genes between planarian neoblasts and schistosome mother sporocysts. They found that of the 1,579 orthologs showing enriched expression in neoblasts, 96.5% (1,525) of these were also upregulated in sporocysts, indicating potentially critical roles in maintaining stem cell totipotency and differentiation (5) (Figure 1). Many genes required for germinal cell maintenance and proliferation were identified among the 581 transcripts, including vasa-like (*vlg*, Smp_068440), polo like (Smp*_*009600), ago2-1 (Smp*_*179320), and fibroblast growth factor receptor (*fgfr*, Smp_175590) (5). In a subsequent study by the same group, stem cells isolated from *in vitro*-transformed mother sporocysts were transcriptionally profiled (6) using principal component analysis of single-cell transcriptomes. Three major sporocyst stem cell classes were clustered (6) based on their respective markers: κ-cells [that produce transcripts of *nanos-2*^+^*klf* ^+^ (krüppel-like factor)]; φ-cells (that transcribe *fgfrA, B*^+^); and δ-cells that produce transcripts of *nanos-2* and *fgfrA, B* (Figure 1). Activation of key genes in κ-cells essential to somatic stem cell function [*fgfr*A/B; *p53*; *zfp-1* (zinc finger protein)] may lead to the formation of δ-cells, a step thought to be important for generating somatic tissues. Downregulation of *nanos*-*2* and activation of *hesl*-*2* (a *hes* family transcription factor) in δ-cells appears to cause the specification of φ-cells, which are involved in many transitory larval structures, such as the tegument of the sporocyst and in the cercarial tail (6). Thus, these cells were checked throughout intramolluscan development by measuring the expression levels of *fgfrA* and *nanos*-*2* (6). During differentiation of daughter sporocysts in mother sporocysts, the φ-cells were observed subjacent to the outer margin of the mother, and were thus excluded from the differentiating embryonic mass, while δ-cells were identified in large clusters within embryos. The κ-cells, additionally could be observed in extraembryonic tissues as singlets or doublets, indicating their important role in serving as the source of developing embryos (6).

The behavior of each cell type in different *S. mansoni* life-cycle stages indicated that stem cells that originate in embryos of daughter sporocysts are sources of the “germline” cells which appear when schistosomes invade the mammalian host, produce gametes (eggs and sperm), and allow the parasites to reproduce sexually (6). Studies of the development of juvenile schistosomes inside mouse blood vessels identified a novel and schistosome-specific gene (*eled*, Smp_041540) as the earliest marker for the schistosome germline (6). Undetectable in sporocysts, *eled* is highly expressed in juvenile stem cells (neoblasts) and distributed in primordial testes, ovaries, vitellaria, and in the posterior growth zone of reproductive organs (6).

### Stem Cells Isolated From Schistosomula

Upon release by snails into fresh water, mature schistosome cercariae swim until they encounter a mammalian host, which they enter by transdermal penetration. During early intra-mammalian development, schistosome stem cells play vital roles in producing the tegument and the esophageal gland, an accessory digestive organ, which develops before the rest of the digestive system is formed (25). Differentiation driven by stem cells is faster than that which occurs in adults, and this results in the rapid growth and development of juvenile parasites (25). During this transdermal migration, a cercaria transforms into the next life-cycle stage, the schistosomulum. Wang et al. (6) compared the transcriptional profiles of proliferating cells isolated from juvenile schistosomula and sporocysts and found 573 genes that were enriched for cell populations from both stages. These enriched transcripts included stem cell markers of schistosomes (*nanos*-*2, ago2-1*, and *fgfrA*), as well as markers of the cell cycle (*h2a, cyclin B*, and *PCNA*) (5, 13). Using the thymidine analog 5-ethynyl-2'-deoxyuridine (EdU) to stain the S-phase cells (proliferating cells) in the parasite, Wang et al. (6) also detected a small number of δ-cells at the early (22–36 h) post-transformation stage in schistosomula and κ-cells in a later (1 week-old) stage. The transformed schistosomula were collected on the other side of mouse tail-skin biopsies after being exposed to cercariae *in vitro*. This evidence supports the concept that sporocyst-derived stem cells drive the initial proliferation of schistosomula indicating that only dividing cells (including a small number of κ and δ-cells) in schistosomula are transmitted to the mammalian host, and are the likely source of neoblast cells in adult schistosomes (8, 13). The report by Wang et al. (6) implied also that the proliferating cells (stem cells) in juveniles may play important roles both in parasite maturation and gonadal differentiation in the mammalian host.

The emergence of single-cell RNA sequencing (scRNAseq) technology has provided a mechanism to accurately determine gene expression profiles and the activities of individual cells. However, it is still a challenge to define meaningful distinctions between cell types when the differences are subtle, due to the fact only an infinitesimally small amount of mRNA can be collected from a single cell, which may result in inaccurate information. Encouragingly, Tarashansky et al. (7) recently established a computational approach called Self-Assembling Manifold (SAM) to classify cell types and to identify novel populations/subpopulations of *S. mansoni* stem cells isolated from schistosomula retrieved from *S. mansoni*-infected mice at 2.5 and 3.5 weeks post infection. Stem cells of schistosomula were separated into three main populations (7) and one of these was further divided into two subpopulations. The three populations included δ'-cells (*nanos-2*^+^*, fgfrs*^+^); ε-cells (*eled*^+^) and μ-cells which express ubiquitous stem cells markers (e.g., *ago2-1*, Smp_179320; *cyclin B*, Smp_082490), all playing critical roles in controlling stem cell division by regulating stem cell-type specific networks. Activation of *hesl* (a hes family transcription factor and a marker of δ-cells) in δ-cells of sporocysts may lead to δ'-cells in schistosomula (Figure 1) (6) which likely serve as the source of the somatic stem cells in adult parasites. Downregulation of *nanos*-*2* and activation of *eled* in κ-cells of sporocysts may generate ε-cells in schistosomula (Figure 1), which are potentially distributed in gonadal primordia, and subsequently give rise to the germline. However, additional in-depth investigation is needed to more fully understand the precise functions of these different cell populations in schistosomes.

In an effort to provide a better understanding of the cell types and the process of tissue differentiation in the early developmental stage of schistosomula, 2-day old schistosomula, obtained by the *in vitro* transformation of cercariae, were efficiently dissociated into individual live cells and sorted cells, obtained using fluorescence-activated cell sorting, were used for scRNAseq analysis (using droplet-based 10X Chromium technology) (26). By combining the SC3, Seurat and UMAP algorithms, a total of eleven discrete cell clusters were discriminated based on their mRNA expression levels and statistically identified marker genes. Only a single stem/germinal cell cluster (in) was identified (26) in the 2-day old schistosomula. Notably, a novel stem/germ cell marker- calmodulin (cam) (Smp_032950) was recognized in the stage, which is a Ca2+ transporter critical for the miracidium-to-sporocyst transition (27), for sporocyst growth and in egg hatching (28), and is also shown to be expressed in the adult gonads and soma. However, further sub-clustering of this stem/germinal cell population was unsuccessful, presumably due to the low expression level of some genes in most of the cells in this cluster.

### Neoblasts in Adult Schistosomes

Similar to planarians (29), schistosomes (13) also possess a broadly distributed population of neoblasts that are able to self-renew and differentiate into many cell types, and which can develop into different tissues including intestine, muscle and tegument (8, 13, 30). Neoblasts that differentiate into tegumental cells are short-lived and are rapidly turned over to renew damaged tegumental cytons. The tegument of adult schistosomes is considered a rich source of molecules that could be targeted by vaccines and drugs to counteract schistosomiasis (31).

It has been demonstrated that genes of adult *S. mansoni*, down-regulated on day 2 following irradiation, were associated with the schistosome neoblasts (13). To further characterize the long-term consequences of neoblast depletion, Collins et al. (8) compared the gene profiles of parasites on day 2 and day 14 after neoblast ablation; they identified 105 *S. mansoni* genes as delayed irradiation-sensitivity (DIS) genes (8), which were only down-regulated on day 14 post-irradiation. These DIS genes may represent genes which require neoblasts for maintaining their expression, and these DIS genes might be active in cells that are directly related to the differentiation of stem cells, possibly cells in tissues that are replenished from the stem cells. Importantly, many of the proteins encoded by DIS genes are expressed in the tegument (32) including *tetraspanin (tsp)-2* (Smp_335630), *Sm13* (Smp_195190), and *Sm29* (Smp_072190) which have shown promise as vaccines in animal models of schistosomiasis (33). These proteins may perform the key functions of schistosome neoblasts, which are important in replacing tegumental cells lost to turnover (34). As indicated earlier, treatment of schistosomiasis currently relies exclusively on a single drug, praziquantel, leading to concerns about the development of drug resistance. It is well-known that schistosomes can repair surface damage caused by sub-curative doses of praziquantel. It is likely that stem cells are involved in the mechanism of this repair process (19), suggesting a potential strategy for drug development by targeting genes critical for maintaining the integrity of schistosomal stem cells. Indeed, a recent report has shown that treatment with FPL-64176, a putative L-type voltage-gated Ca2+ channels agonist, can lead to tegument disruption in adult *S. mansoni* and inhibition of mitotic activity in somatic stem cells and germ line tissues (35).

To uncover new schistosome targets with therapeutic potential, Collins and his team (36), using large-scale RNAi screening, identified 66 genes required for stem cell survival in adult *S. mansoni* (36). Furthermore, by utilizing advanced scRNA seq technology, the same group described 68 molecularly distinct cell populations in adult worms of *S. mansoni* (37) comprising the majority of all tissues that have been described morphologically in schistosomes, including the reproductive and nervous systems. The work additionally revealed a lineage of somatic stem cells responsible for producing and maintaining the schistosome gut (the primary organ responsible for digestion of host blood, a major energy source essential for parasite survival). Furthermore, the study showed that a homolog of hepatocyte nuclear factor 4 (*hnf4*), likely responsible for gut maintenance, blood feeding and in inducing egg-induced pathology *in vivo*, was expressed in this gut lineage (37).

Classical studies of cell proliferation in blood flukes have focused on the reproductive system of adult worms (38), given the critical dual role of eggs in schistosome transmission and in the immunopathology induced by ova trapped in host tissues. Uniquely amongst the Platyhelminthes, female adult schistosomes live partially enclosed within a specialized gynaecophoric canal on the ventral surface of the male worm's body; the growth and sexual maturity of female worms are heavily reliant on continuous pairing with male worms to fuel the maturation of their reproductive organs (39). Maturation of the vitellaria, which produce cells that form egg shells and are packaged into the egg with the zygote, is highly dependent on the presence of the male. Vitelline tissues regrow when unpaired females start to mate again with males, possibly due to the activation of vitellarium-specific stem cells (S1 cells) (40), although the relationship between the S1 cells and neoblasts remains unclear. Furthermore, by comparing the gene profiles of ovaries and testes isolated from paired and unpaired schistosomes, Lu et al. (41) demonstrated that pairing-induced processes within the gonads are intimately involved in stem cell-associated and neural functions. Several stem cell genes including *polo-like kinase1* (42), *vasa-like genes1–3* (43), and *fgfrA* and *B* (13, 44) were shown to be extensively associated with gonadogenesis and gametogenesis. It was also demonstrated that specific stem cells located in the primordial ovaries and vitellaria of unpaired schistosome females were able to differentiate into oocytes and vitellocytes after the females were mated with males (39). In a study aimed at further understanding how the stem cells in the reproductive organs respond to external cells producing differentiated progeny (i.e., oocytes or vitellocytes), Wang et al. (45) identified an uncharacterized gene (Smp*_*248100) sharing sequence similarity with members of the NR (nuclear receptor) family of ligand-activated transcription factors; this gene was exclusively expressed in the vitellaria of both mature and immature females (45). In immature females, Smp*_*248100 is expressed in the S1 cells of the primordial vitellaria (46), indicating its key role in the differentiation of these cells (39). Given that stem cells may represent attractive anthelminthic targets, quantification of proliferating schistosome stem cells in gonads (testes, ovaries) and parenchyma has been used to assess the effects of harmonine, an antimicrobial alkaloid, as a novel antischistosomal drug (47).

## Recent Improvements in Schistosome Cell Culture

The majority of current physiological, cellular, and functional studies of individual gene products of multicellular platyhelminth parasites have been undertaken using non-ideal heterologous expression systems utilizing bacteria, yeast, and insect and mammalian cells. The lack of self-renewable parasitic flatworm cell lines represents a significant barrier for translating genomics advances into gene-level functional investigations in single parasite cells. This has hindered the development of new diagnostics and interventions necessary for controlling the spread of the diseases caused by the more pathogenic flatworms such as the *Schistosoma* spp. The recent single cell RNA-seq studies with *S. mansoni* and the characterization of different stem cell populations, however, provide an important database to develop immortalized cell lines.

In early attempts to develop a suitable *in vitro* cell culture system for studies on the cellular and molecular biology of *S. mansoni*, Bayne et al. (48) developed proliferating cell lines from tissue fragments originating from *in vitro*-derived mother sporocysts. Co-culturing these cells with host snail ganglia maintained their viability for several months and, during this period, differential survival of individual cell types was observed, although indefinite propagation was not achieved. Subsequently, the same group (49) improved on the *in vitro* technique by culturing cells prepared from juvenile worms (obtained 12–21 days after cercarial infection) and mother sporocysts; these cells exhibited long term viability (up to 8 months) with limited cell proliferation. In addition to the work with *S. mansoni*, a number of studies have also been undertaken to develop improved *in vitro* cultivation methods for *S. japonicum* with cells derived from miracidia, cercariae, and egg-laying adult worms passaged up to the 6th, 5th, and 8th generations, respectively (50), however, the particular types of these cells were not clearly identified. Hahnel et al. (51) succeeded in isolating pure and intact testis and ovary cells from adult schistosomes, providing a basis for cell isolation and future development of cell lines. A very recent study has been shown that extracellular matrix is a regulator of the stem cell compartment in the planarian *Schmidtea mediterranea*, playing a particularly important role in stem cell growth (52). It might be interesting to marry this strategy into schistosome stem cell culture, so as to enhance culture conditions and potential proliferation of stem cell lines. Nevertheless, the further optimization of culture conditions will be critical to open up new avenues for obtaining immortalized schistosome cell lines.

## A Way Forward for Developing Self-Renewable Flatworm Cell Lines–The *Echinococcus* Model

Klaus Brehm and his group at the University of Wuerzburg in Gernany are successfully using the tapeworm Echinococcus multilocularis as a model to generate a highly enriched stem cell system from immortal metacestode larvae (53), currently the only parasitic helminth cell line available. The establishment of successful cultivation of Echinococcus spp. stem cells points the way forward for elucidating different developmental transitions and processes in these cestodes. The approach could be exploited for studies of other flatworm taxa including improving the *in vitro* culture of both schistosome stem cells and those of other trematodes such as Fasciola hepatica, given that the growth of this liver fluke involves the division of cells resembling stem cells (54).

The metacestode of *E. multilocularis* has a unique process of development, growing continuously as a vesicular mass which infiltrates the tissues of the intermediate rodent host, generating multiple protoscoleces by asexual budding (53, 55). This unique proliferation potential indicated the existence of stem cells that are totipotent with the ability for extensive self-renewal (53). Successful *in vitro* cultivation of larval *E. multilocularis* provided a mechanism for generating mature metacestode vesicles under laboratory conditions in a manner akin to the oncosphere-metacestode transition during a natural infection (55). The formation of protoscoleces within metacestode vesicles and cysts of *E. multilocularis* and the related *E. granulosus* is induced by neoblasts (55). Similar to the neoblasts of free living flatworms, germinative cells were recognized as the only source of proliferating cells in *E. multilocularis* (53), presumably driving the growth of the larval vesicles. Co-culture of *E. multilocularis* cells with host feeder cells, which can produce growth factors necessary for proliferation, was able to significantly improve the growth of isolated parasite cells (56). Recent *in vitro* study showed that fibroblast growth factor (*fgf* ) signaling, activated by the binding between human FGF and the tapeworm FGF receptors (which are important stem cell markers), is responsible for stimulating the formation of metacestode vesicles from neoblasts and supports metacestode growth (57). Albani et al. (58) subsequently developed a long-term *in vitro* culture system for *E. granulosus* germinal cells, isolated from the germinal cyst layer, that were able to grow beyond 100 passages (58, 59); after 1 month of culture, two distinct cell populations, attached cells and suspended cells, developed (58). The suspended cells were shown to clump forming, after 2–3 months in culture, a tridimensional formation of cell clusters with a central cavity surrounded by different cell types merged in an extracellular matrix without splitting (58). Cyst-like structures were evident in the livers of mice at 12 months following injection of these suspended cells (58). These findings imply that the Echinococcus stem cell system may be applicable for identifying new targets for the development of anti-echinococcosis drugs. It is notable that very limited effects on echinococcal germinative cells were observed after they were treated with albendazole (53); this could explain the high recurrence rates occurring in echinococcosis patients after albendazole treatment (60). Furthermore, it emphasizes the potential value of targeting *Echinococcus* spp. stem cells in the search for novel therapeutics against echinococcosis (61).

## Conclusion

Stem cells ensure schistosomes are able to thrive during their complex multi-generational life cycle and they also promote parasite longevity by playing key roles in pathogenesis in definitive hosts (14). It is important to dissect the roles that schistosome stem-like cells play in each of the parasite's life stages and how they drive differentiation processes throughout the complete lifecycle so that these blood flukes can thrive in the different environments they encounter. Overall, the three take-home messages covered by articles considered in this review are:

1. Schistosome stem cells play key roles in different life cycle stages, including the germinal cells of sporocysts involved in asexual reproduction in molluscan hosts and the neoblasts of adult worms involved in sexual reproduction in definitive mammalian hosts. Their study will enable the development of a better understanding of the stem cell heterogeneity driving the propagation of the schistosome life cycle. In addition, the common functions and make-up of germinal cells and neoblasts indicate a similar network may regulate these cells in the different developmental stages. Coupled with this information, future research aimed at characterizing the roles of the different stem cell populations, could provide a pathway for developing new interventions to treat and prevent schistosomiasis.
2. Uncovering novel genes, predominantly expressed in stem cells, and deciphering the roles they play in controlling reproductive development and parasite survival may lead to the identification of critical anti-schistosome drug and/or vaccine targets. This is because the pathogenesis of schistosomiasis in mammalian hosts is due to the regenerative capacities of schistosome stem cells whereas adult pluripotent stem cells drive long-term homeostatic tissue maintenance in these long-lived flatworms. Targeting proteins produced by stem cells would likely impact on schistosome defense mechanisms with potential for preventing the transmission of schistosomiasis.
3. The future establishment of self-renewable resources will provide critical biological materials for translating genomics advances into gene-level functional investigations in single parasite cells. This is important for studying individual gene function, and for optimizing genome-editing tools, such as CRISPR/Cas9 editing in schistosomes recently developed by our laboratory (62) and others (63, 64); this can provide a blueprint for developing self-renewable cellular resources from the parasitic platyhelminth flatworms at large. Such resources will be key in the development of novel treatments and immunological intervention to prevent schistosomiasis transmission by targeting the early stages of infection as stem cells are intimately linked to the earliest developmental stage of the germline.

## Author Contributions

HY wrote and revised the manuscript. DPM, MKJ, and DJW contributed to the original submission, revision of the manuscript and its editing. All authors approved the final submitted version of the article.

## Conflict of Interest

The authors declare that the research was conducted in the absence of any commercial or financial relationships that could be construed as a potential conflict of interest.
